# Effects of visual information presented by augmented reality on children’s behavior

**DOI:** 10.1038/s41598-020-63820-z

**Published:** 2020-04-22

**Authors:** Nobu Shirai, Lisa Kondo, Tomoko Imura

**Affiliations:** 1 Department of Psychology, Faculty of Humanities, Niigata University, 2-8050 Ikarashi Nishi-Ku, Niigata, 950-2181 Japan; 2 Department of Psychology, Faculty of Integrated Arts and Social Sciences, Japan Women’s University, 1-1-1, Nishiikuta, Tamaku-ku, Kawasaki, 214-8565 Japan

**Keywords:** Psychology, Human behaviour

## Abstract

The effects on children’s behavior of visual information presented by augmented reality (AR) were investigated. A human-like AR character was presented standing in one of two physical pathways to children aged 5–7 years old and 8–10 years old before they completed a filler task. After the task, the children were required to walk through one of the two pathways to obtain a reward. Both the 5–7- and 8–10-year-olds chose the pathway that was not associated with the AR character more frequently than the pathway that was. Subsequently, adult participants tested in a similar manner showed no significant bias in pathway selection. Taken together, these results suggest that the presentation of an AR character within the present experimental setting affected the behavior of children aged from 5–10 years but not that of adults. The results are discussed in the context of developmental changes in sensitivity to insubstantial agents (e.g., imaginary companion), the reality of information displayed by AR technology, and differences in the methods of AR presentation (e.g., hand-held devices vs. head-mounted devices).

## Introduction

Technologies that expand the human sensory experience, such as virtual reality (VR) and augmented reality (AR), have been rapidly popularized during the last decade. In general, VR completely replaces sensory information from the natural environment with artificial information; e.g., fully masking one’s vision with computer-generated visual stimuli using a head-mounted display. In contrast, AR superposes artificial sensory information on information from the natural environment; e.g., overlaying a computer-generated avatar onto a scene of the real world in real time using a camera and smartphone display.

In today’s society, opportunities to be involved with these types of technology are increasing for adults as well as children. A variety of consumer VR headsets have been released by many companies at reasonable prices, and some of these VR headsets are designed for young children. For example, children 7 years of age and older are allowed to use the Nintendo Labo VR Kit^1^. Furthermore, against a background of the worldwide spread of personal portable electronic devices (PED), including smartphones and tablets, a variety of applications and games have adopted AR for PED users, resulting in a variety of uses for these technologies and the involvement of many people. For example, Pokémon GO, which is one of the most popular AR games, has been downloaded more than 800 million times^2^.

The present study investigated whether AR experiences would influence children’s behavior. Previous research has shown that information presented by AR technology has considerable effects on the mental and behavioral responses of human adults. For example, adult observers subjectively report more entertaining and realistic experiences under a gaming situation with an AR-generated avatar than with a similar avatar displayed as a 2-D- or 3-D-modeled cartoon character^3^. Additionally, adult observers watching a human-like AR avatar via a head-mounted device tend to control their walking path to avoid the space in which the AR avatar appears, just as they do for a real human model^4^. Although many studies have demonstrated the advantages of using AR for pragmatic purposes, such as the potential merits of AR in educational situations^5–8^, few studies have investigated the effects of AR on children’s behaviors using a scientific approach. Therefore, the present study investigated whether visual information displayed by AR, such as a human-like agent, would have any significant effect on children’s behavior under experimental situations.

Although the full range of effects of AR agents on children’s behavior remains unknown, Piazza *et al*.^9^ found that the verbal suggestion of the presence of an insubstantial agent alters behavior in children aged 5–6 years and 8–9 years^9^. In that study, children were instructed to engage in a very challenging game task in which successfully putting a sticky ball in the central area of a target earned them a reward. However, it was essentially impossible to succeed in this task without cheating because the children were required to follow three stringent rules during the game: throw the ball from a position 6 feet away from the target, use the non-dominant hand to throw the ball, and stand with their back to the target when throwing the ball. Prior to engaging in the task, the children were divided into three groups according to the task conditions: with an invisible agent, with adult supervision, and with no supervision. Under the invisible agent condition, each child was told, “An invisible girl will stay with you in this room and observe your behaviors.” They then engaged in the task while remaining alone in the room. Under the adult supervision condition, each child engaged in the task under the supervision of an adult, who stayed in the room and observed the child’s behavior during the game. Under the no supervision condition, the children were given no specific instructions about an observer, and they engaged in the game task in the room alone. The latency to full cheating behavior during the game task (i.e., walking toward the target and directly putting the ball on the target with their hand) was measured as the dependent variable. Children’s cheating behavior was reduced in both the 5–6- and 8–9-year-old groups by the presence of an actual human observer as well as by an invisible agent such that the mean latency to full cheating behavior was significantly longer under the invisible agent and adult supervision conditions than under no supervision.

Based on the findings of Piazza *et al*.^9^, our research group conducted a preliminary study to investigate the effects of an AR character on children’s behavior^10^ (poster presentation at an international conference. A digital copy of the poster is available at ‘https://nyu.databrary.org/volume/1112’). In our study, 5–7-year-old and 8–10-year-old children engaged in the same task as that used by Piazza *et al*.^9^ under the following experimental conditions: (1) the AR agent condition, in which a three dimensional (3D) AR cartoon agent were shown to the children prior to the game; (2) the TV agent condition, in which the same cartoon agent as used for the AR condition was shown to children on a TV screen prior to the task; and (3) the no agent condition, in which no agent was shown to children. The results indicated that, in the younger group, the mean latency to full cheating behavior was shorter under the AR than under the other two conditions; thus, that the younger children’s cheating behavior appeared to be promoted by the pre-presentation of the AR character. Although the reasons for this finding were unclear, these results imply that visual information presented by AR has some impact on young children’s behavior.

Although the preliminary study conducted by our research group demonstrated that visual information presented by AR potentially affects children’s behaviors, the study had several limitations that should be noted when interpreting the results. First, the preliminary study included six groups (two age groups × three agent conditions) with 12 children in each group; thus, a total of 72 children took part in the study. Although the sample size per group was comparable to that of Piazza *et al*.^9^ (9–14 children per group), this number is insufficient to generalize the results (cf.^11^). The second issue is associated with the validity of the game task used in the preliminary study. Only 10 of the 72 participants exhibited full cheating behaviors in the game task in the preliminary study, whereas 18 of 67 participants showed full cheating behaviors in the study by Piazza *et al*.^9^ (Important note: the number of children who showed full cheating behaviors was not explicitly reported by Piazza *et al*.^9^ but was calculated based on the reported rate of children who showed full cheating and the reported sample size in each experimental group). Thus, the number of children who exhibited full cheating behavior in our preliminary study was relatively small. This discrepancy implies that the preliminary findings might have been biased by the behavior of only a few participants. More specifically, only two of 24 children engaged in full cheating behavior under the no agent condition in the preliminary study, whereas 10 of 24 children did so in the study by Piazza *et al*.^9^ Furthermore, differences in responses to this particular task may exist among various cultures and societies; e.g., Japanese children may be less responsive to the game. Taking the limitations of the preliminary study into consideration, the present study evaluated the effects of visual information provided by AR on behaviors in children using a simple experimental task with a larger sample size.

## Experiment 1

### Method

#### Ethics statement

The present study was approved by the Ethics Committee for Human Research at Niigata University, and all experiments were conducted in accordance with the principles of the Declaration of Helsinki.

#### Participants

Experiment 1 included 24 younger children (12 girls, mean age = 6.32 years, standard deviation [SD] = ±0.87, range = 5.07–7.96 years) and 24 older children (11 girls, mean age = 9.37, SD = ± 0.98, range = 8.21–10.99 years). Three additional children participated in the experiment but were excluded from the final sample due to the following experimenter errors: (1) the experimenter forgot to ask the parents about the AR experiences of two children and (2) the experimenter wrongly recruited and tested a non-naïve child who had participated in a preliminary study by our research group^10^. The parent(s) of each child provided written informed consent prior to the experiment and confirmed that the participants had no visual deficits or known history of any developmental disorder.

#### Apparatus and stimuli

**Figure 1:**
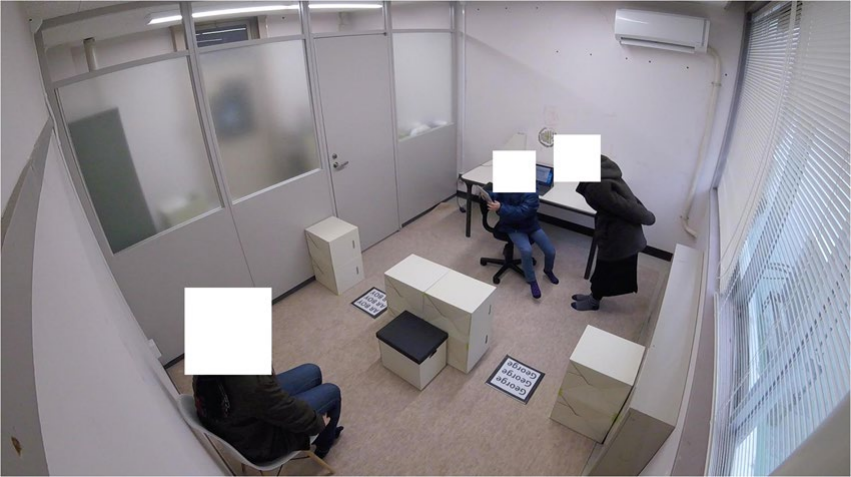
Snapshot of the experimental room taken by the hidden camera attached to the upper corner of the experimental room. In area 1 (the upper-right area of the picture), a participant was looking for the AR agent using the tablet device. A main experimenter was standing near the participant and confirmed whether the participant appropriately observed the AR agent. An assistant sat on a chair in area 2 (the lower-left area of the picture) and observed the participant without speaking (Note: informed consent regarding the publication of this picture in an online open-access journal was obtained from the people appearing in this figure).

**Figure 2:**
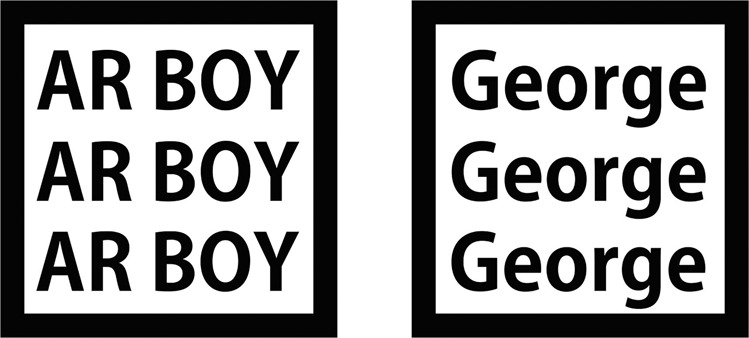
Examples of the AR marker sheets. In each experiment, one of the two markers was used as the marker for the AR character (an AR character appeared on the marker), and the other was used as a marker without the AR character (no AR character appeared on the marker). The marker on which the AR character appeared was counterbalanced across participants.

All experiments were conducted in a room (3.8-m width × 2.7-m depth, Fig. 1) with standard illumination; the room was divided into two areas: area 1 (A1) and area 2 (A2). In A1, a table and chair that were used to perform a dummy task were placed facing toward one of the side walls of the room. In A2, a chair was placed near the other side wall of the room where as assistant (see the Procedure section for details) sat during the experiment. A1 and A2 were separated by block barriers (55 cm in height) placed approximately across the middle of the room; these were arranged so as to leave two open aisles (60 cm in width) that could be used to come and go between A1 and A2. Two marker sheets (30 cm × 30 cm, Fig. 2) that displayed the AR character and had different figures on them were fixed to the floors in the aisles; one of the markers was placed in the aisle on the door side of the room, and the other marker was placed in the aisle on the window side of the room. The position of the markers (i.e., which marker was placed in which aisle) was counterbalanced across participants. A hidden camera (HERO3+, GoPro, Inc.; San Mateo, CA, USA) was placed in an upper corner of the room to record participants’ behaviors during the experiments (Fig. 1).

A tablet device (iPad 4th generation, 32 GB model, Apple, Inc.; Cupertino, CA, USA) displayed the AR character to the participants using a custom application developed by the Unity 3D game engine (Unity ver.5.6.3p1, Unity Technologies SF, Inc.; San Francisco, CA, USA) with an AR library for Unity (Vuforia 6.2, PTC, Inc.; Needham, MA, USA). Using the application on the tablet, the participants could see the AR character, which was a 3D-modeled cartoon avatar of a boy, superimposed on the real scene. When a participant turned the back camera of the tablet toward an AR marker on the floor of an aisle, the AR character appeared on the tablet screen, just as if the character were standing on the AR marker. The height of the AR character in the simulated space that was superimposed on the real environment was approximately 80 cm.

The avatar of the AR character was “Male3_shortHairNGAvatar” and was adopted from the “Cute Male 3” package (provided by TurkCheeps; https://assetstore.unity.com/packages/3d/characters/cute-male-3-18090), which is distributed on the Unity asset store (https://www.assetstore.unity3d.com). Model data for the actions of the avatar were adopted from the “Taichi Character Pack” package (provided by GAME ASSET STUDIO, https://assetstore.unity.com/packages/3d/characters/taichi-character-pack-15667) and implemented in the application by a built-in animation control system of the game engine for the 3D avatar (Unity, Mechanim). Variations in the actions of the avatar included putting his right hand on the back of his head and turning his head to the right side (‘idle_00’ of the data package), standing straight (‘idle_01’), looking around to the right and left and then looking down and kicking the ground gently with his right foot (‘idle_10’), looking at the participant and waving his right hand (‘greet_02’), and scratching his head with his left hand (‘scratchhead_00’). These actions were serially performed in accordance with a preprogrammed sequence (starting with ‘idle_00’ followed by ‘idle_01’, ‘idle_10’, ‘greet_02’, ‘scratchhead_00’, and ‘idle_01’). One stroke of the action sequence was approximately 16 sec, and the sequence ran repeatedly during use of the application. Once the application was started, the action sequence automatically started and continued running regardless of the avatar’s visibility. Thus, even when the avatar was invisible (e.g., when a participant was not turning the tablet’s camera toward the AR marker so the AR character was not appearing on the tablet screen), the action sequence continued in the background in real time.

#### Procedure

**Figure 3:**
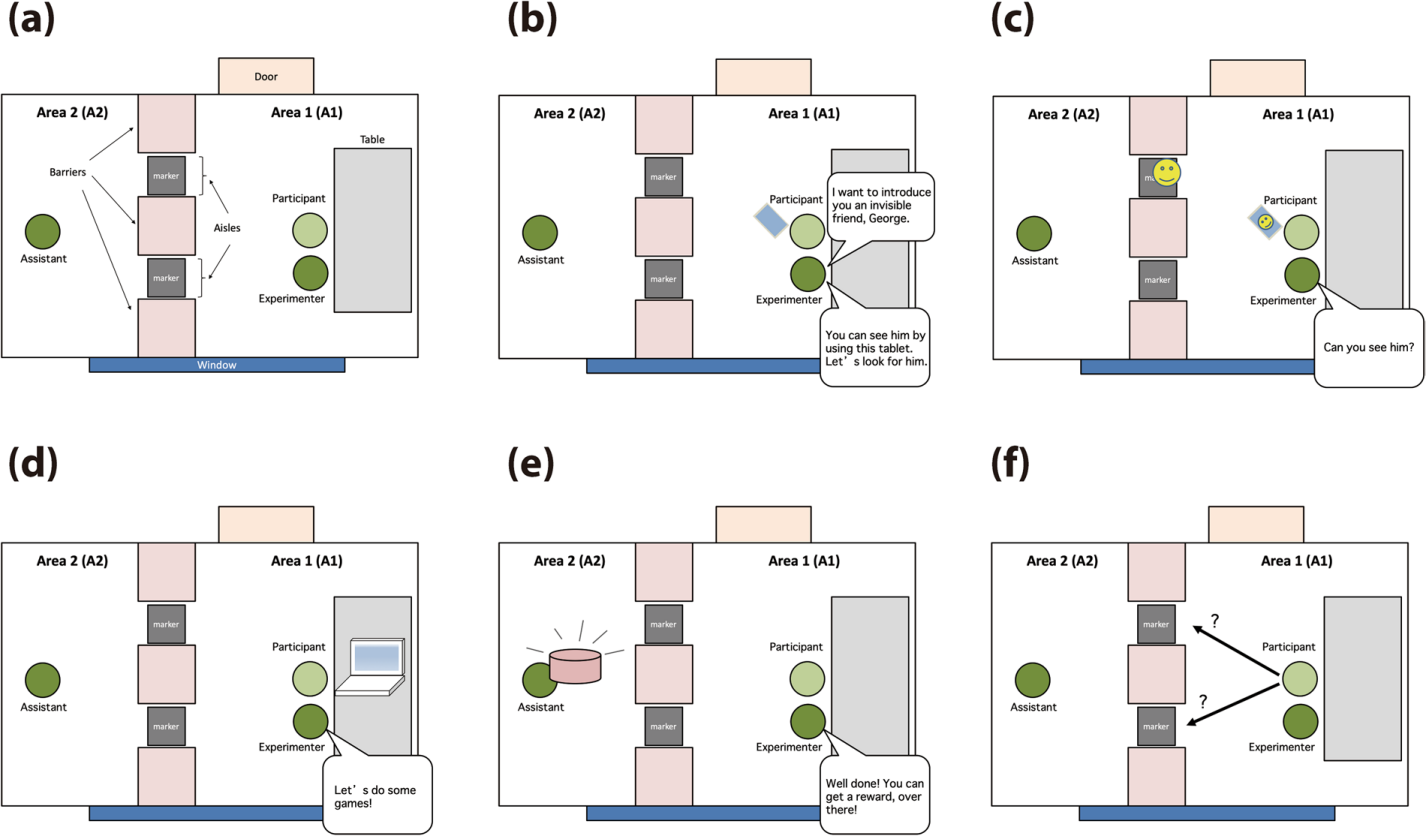
Flow chart of the experimental procedure. (**a**) The main experimenter escorted a participant into A1 of the experimental room, while an assistant was already sitting in a chair in A2. (**b)** The experimenter introduced the invisible agent “George” to the participant and (**c**) urged the participant to look for George using a tablet device. (**d**) After confirming that the participant observed the figure of George on the tablet’s screen, the filler task was begun. (**e**) After the filler task, the experimenter told the participant to go to A2 to get a reward. (**f**) The participant’s aisle choice to get to A2 was recorded.

The entire flow of the experimental procedure is illustrated in Fig. 3. Before a participant and the main experimenter (experimenter) entered the room, an assistant experimenter (assistant) sat in a chair placed in A2. Then, the experimenter escorted a participant into the experimental room and led that individual to a chair in front of the table in A1. The experimenter introduced the assistant to the participant using the following phrase: “This is [the assistant’s name] who helps me to record this experiment”. Then, the assistant lightly bowed her/his head in a friendly manner without any verbal response. During the experiment, the assistant made mild, calm, and friendly expressions but never stood up from the chair or spoke to the participant or the experimenter.

The experimenter then told the participant to, “Have this chair, please”, to gently urge the participant to sit in the chair. After the participant sat down in the chair, the experimenter stood at either the right or left side of the participant (the experimenter’s standing position did not change during a given trial and was counterbalanced across participants) and began to talk about “an invisible friend” who was somewhere in the experimental room as follows: “Today, I would like to ask you to cooperate in a game for our experiment. But, before we start to do an experiment, I want to introduce a friend to you. His name is George. Because he is so shy about showing himself to others, he usually makes himself invisible. So, we can’t see him with our eyes. But he is surely in this room and watching us right now.” Then, the experimenter handed a tablet to the participant and gave additional instructions about how to visually find George as follows: “He is actually friendly and loves to show himself on the tablet’s camera. So, you can see George by using this tablet device. Let’s look for George using this tablet.” Next, the experimenter gently urged the participant to look at both of the AR markers on the floor. The AR character appeared on either the AR marker on the window side or the one on the door side of the room (Fig. 3c, door side); the location where the AR character appeared was counterbalanced across participants. If a participant seemed to have difficulty searching for George or was not voluntarily searching for George, then the experimenter pointed to the marker at which the AR character had appeared using her hand and told the participant that, “I think … he is about here,” to help the participant find George. If the participant still failed to find the AR character, then the experimenter would place her hands on the tablet and gently move it to help the participant find the AR character. After confirming that the participant had successfully observed George on the tablet screen, the experimenter asked the participant to engage in a filler task (introduced as, “O.K. It’s time to start the game. Let’s start now!”), which was a computer-based simple size discrimination task. After a brief set of instructions and several practice trials, the filler task was started on the table in A1 and lasted for approximately 2 min. When the participant had finished the filler task, the experimenter said to the individual, “Well done! Now, you finished the game. [the assistant’s name] will give you something special. Go over there and get a gift!”; then the participant moved on to A2 to get a reward (small toy, pen, eraser, sticker, etc.) from the assistant. The assistant held a box at about chest level and showed the rewards to the participant. At this point, the assistant was allowed to talk gently to the participants (e.g., “Please come over here and get a gift!”) without any gestures if a child hesitated to go into A2. The only pathways to pass through the barriers between A1 and A2 were the abovementioned aisles; thus, each participant was forced to choose one of the two aisles to get to A2. Each participant’s pathway selection (choice of an aisle) was recorded and used as the dependent variable. The typical length of the period from the end of observing the AR character to the moment at which the participant passed through the barrier was approximately 3.5 min. However, it should be noted that the reported typical time length is a rough estimation. Due to limitations of the video recordings in terms of the viewing angle and resolution, it was not possible to confirm the exact moment at which the AR character disappeared from the tablet screen and/or the moment at which the participant looked away from the tablet screen.

After the experiment was completed, the parents were interviewed about their child’s experience with AR technology prior to the day of the experiment. If a parent reported that the child had experienced AR technology (e.g., game, application for selfie, etc.) at least once, then that child was categorized as an ‘AR experiencer’.

The position of the experimenter (either at the window or door sides), the AR character (either at the window or door sides), and the type of marker for the AR character (either ‘ARBOY’ or ‘GEORGE’, see Fig. 2) were completely counterbalanced across each age group. That is, there were eight combinations of the position for the experimenter, the position of the AR character, and the type of marker for the AR character; these eight combinations were assigned to the 24 children in each age group.

## Results

**Figure 4:**
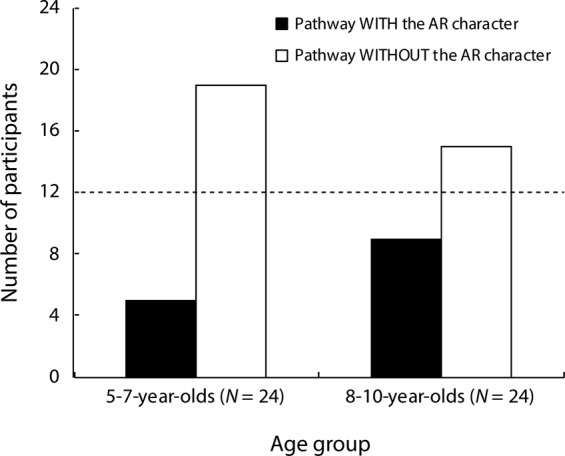
Results of Experiment 1. The bars on the left and right sides of the graph display the results for children aged 5–7 years old and 8–10 years old, respectively. The black and white bars represent the number of participants who selected the pathways with and without the AR character, respectively. Individual data from Experiment 1 are available at ‘https://nyu.databrary.org/volume/1111’.

In Experiment 1, 19 of 24 younger children (5–7-year-olds) and 15 of 24 older children (8–10-year-olds) chose to pass through the barrier using the aisle that was not associated with the pre-presentation of the AR character (Fig. 4). These results appear to be consistent with those of our preliminary study^10^ showing that the effects of information presented by AR technology on children’s behaviors are more remarkable in younger children (5–7-year-olds) than in older children (8–10-year-olds). However, a two-tailed Fisher’s exact test revealed that the number of children who chose the aisle without the AR character compared to the number of children who chose the aisle with the AR character did not significantly differ between the younger and older groups (*p* = 0.341, *φ* = 0.183), which indicates that the tendency of pathway selection did not differ between the younger and older children. It is possible that prior experience with AR technology might have affected the pathway selection of the children and, thus, all participants (N = 48) were divided into two groups based on previous AR experience (AR experiencer: N = 26, 10 girls, mean age = 8.10 years, standard deviation [SD] = ±2.00, range = 5.07–10.99 years; non-AR experiencer: N = 22, 13 girls, mean age = 7.58 years, standard deviation [SD] = ± 1.52, range = 5.11–10.47 years) to compare the tendency for pathway selection between these two groups. Eighteen of 26 AR experiencers and 16 of 22 non-AR experiencers selected the aisle without the AR character and a two-tailed Fisher’s exact test revealed that the tendency for pathway selection did not significantly differ between the AR experiencers and non-AR experiencers (*p* = 1.000, *φ* = 0.038). These results indicate that prior experience with AR technology had no effects on the pathway selections of children in the present experiment.

The results of the present analyses assessing the participants’ ages and previous experiences with AR technology suggest that there was no valid reason to divide the participants into subgroups. Thus, the data of the younger and older children were combined into a single group (N = 48) for further statistical analyses. Two-tailed binomial tests were conducted to examine whether the number of participants who chose the pathway with the AR character differed significantly from the number of participants who chose the pathway without the AR character and revealed that significantly (*p* < 0.006, Cohen’s *g* = 0.208) more participants selected the pathway without the AR character (N = 34) than that with the AR character (N = 14). Thus, the results of Experiment 1 suggest that the 5–10-year-old children tended to avoid the pathway where the AR character pre-appeared and that the visual information presented by AR technologies may have had a significant impact on behavior in children in this age group.

## Experiment 2

Experiment 1 demonstrated that the presentation of an AR character had a significant effect on pathway selection in children between 5 and 10 years of age. However, it remains unclear whether similar effects would be observed in older individuals, such as adults, using the present experimental setting. Previous studies have reported that AR-generated information has considerable effects on the behavior of human adults^4^. It should be noted that the observers’ responses in that previous study were assessed while they watched the AR avatars in real time whereas the present experiment examined participants’ responses after they had watched the AR character. It will be important for future studies to determine whether the significant effects of AR-generated information differ based on the timing of the information being presented by AR. Additionally, Bering *et al*.^12^ reported that adult participants change their behaviors based on the suggested presence of an invisible and insubstantial agent, such as the idea of a ghost in the experimental room, and that this inhibits cheating behaviors during an experimental task. Thus, it is plausible that adult participants would exhibit behavioral tendencies similar to those of the children in Experiment 1, even though they had to make their pathway selection after watching the AR character. Therefore, Experiment 2 examined whether the presentation of an AR character would influence pathway selection in adults using almost the same experimental procedure as Experiment 1.

## Method

### Participants

Experiment 2 included 24 adult participants (nine females, mean age = 21.75 years, SD = ±2.01, range = 18.99–28.64 years); one participant was excluded from the final analyses, because that individual recognized the actual aim of Experiment 2. However, based on verbal reports made during the debriefing session after Experiment 2, none of the other participants had recognized the actual aim of Experiment 2. Although written informed consent was obtained from all participants prior to Experiment 2, the consent forms were updated and reconfirmed soon after the experiment because the procedure for Experiment 2 contained a mild deception, described below.

### Apparatus, stimuli, and procedure

The experimental setting for Experiment 2 was almost identical to that for Experiment 1 except that a different cover story was used for showing the AR character to the participants. It would be inappropriate to apply the children’s story from Experiment 1 (i.e., “An invisible friend is watching you in this room and you only can see his figure via AR equipment”) to the adult participants. Hence, the cover story was modified to be more realistic and pragmatic for adult participants.

Briefly, the main experimenter (experimenter) escorted a participant to the experimental room and led that individual to a chair in front of the table in A1. Another experimenter (assistant) was already sitting in a chair in A2, preparing a visual pseudo-experiment on a laptop computer. Then, the experimenter introduced the assistant to the participant (e.g., “This is [the assistant’s name] who will run today’s experiment”) and the assistant greeted the participant (e.g., “Hello. Nice to see you!”). Next, the experimenter told the participant to, “Have this chair, please”, to gently urge the participant to sit in the chair. After the participant was seated, the experimenter stood by either the right or left side of the participant (the standing position of the experimenter did not change during each trial and was counterbalanced across participants) and provided instructions for the pseudo-experiment. Next, the experimenter asked the participant to go to A2 to engage in the pseudo-experiment. Then, the assistant suddenly said “Oh, I am having trouble with a computer program for the experiment. I will have it fixed soon, so please wait there for a moment”. The experimenter confirmed this story and told the participant “OK, now we need to wait for a while. I would like to offer you a chance to try our new experimental material, which is under development: an AR character for a child study. Please try it; we welcome your comments about the character”.

Next, the experimenter handed a tablet to the participant, and asked him/her to look at both AR markers on the floor. The AR character appeared either at the AR marker on the window side or at the one on the door side; the side was counterbalanced across participants. After the participant successfully observed the AR character on the tablet screen, the assistant said to the participant “The problems have been fixed. Please come over here and let’s start the experiment.” The pathway selected by the participant for the transition from A1 to A2 was recorded and used as the dependent variable. The typical length of time between the end of observing the AR character and the moment at which the participants passed through the barrier was less than 1 min. Soon after the participant reached the assistant, a debriefing that described the true objective of Experiment 2 was provided to each participant, completing the experiment.

The position of the experimenter (either at the window or door sides), the AR character (either at the window or door sides), and the type of marker for the AR character (either ‘ARBOY’ or ‘GEORGE’, see Fig. 2) were completely counterbalanced across the participants in the same manner as in Experiment 1.

## Results

**Figure 5:**
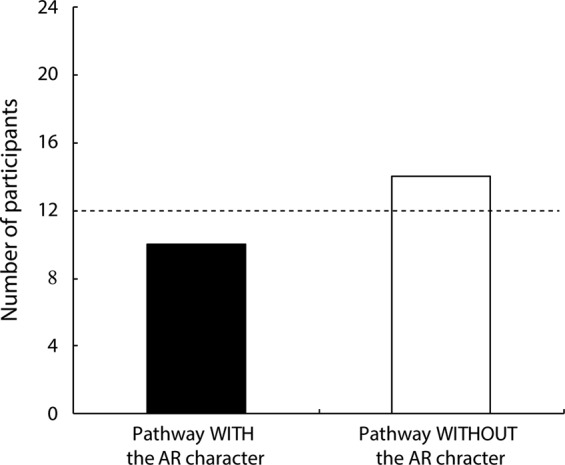
Results of Experiment 2. The black and the white bars represent the number of participants who selected the pathways with and without the AR character, respectively. Individual data from Experiment 2 are available at ‘https://nyu.databrary.org/volume/1111’.

In Experiment 2, 14 of 24 adult participants chose to pass through the barriers using the aisle that was not associated with the pre-presentation of the AR character (Fig. 5). A two-tailed binomial tests revealed no significant difference between the number of adult participants who selected the pathway without the AR character and the number who chose the other pathway (p = 0.541, Cohen’s *g* = 0.083). These results imply that the presentation of an AR character had no effect on the behavior of adults in the present experimental setting.

## General Discussion

The primary aim of the present study was to investigate whether visual information displayed using AR technology would have any effects on children’s behavior. In Experiment 1, 5–7-year-old and 8–10-year-old children were shown an AR cartoon character, pre-presented on the floor in one of two aisles. When the children were required to pass through one of these aisles, they more frequently chose the aisle not associated with the pre-presentation of the AR character than that associated with the AR character. Although this tendency in pathway selection seemed to be more remarkable in the younger children than the older children (see Fig. 4), this difference was not statistically significant. In Experiment 2, adult participants were evaluated in a similar experimental situation, and they did not exhibit a significant preference in pathway selection between the aisles with and without the pre-presented AR character. Taken together, these results imply that the effects of visual information presented by AR (e.g., a human-like cartoon character) had a significant effect on the behavior of children (approximately 5–10 years of age) but not on adults under the specific experimental setting used in the present study.

It is possible that the presence of an AR character had a significant effect on pathway selection only in the children because young children tend to have a higher sensitivity to insubstantial agents than older individuals. For example, young children often engage in steady communication with imaginary companions (e.g., talking with an invisible friend) or characters that are embodied in inanimate objects (e.g., treating dolls or stuffed toys as animated characters). Additionally, it has been reported that the experience of having imaginary companions by Japanese children peaks at approximately 4 years of age and then significantly declines beginning at 7–9 years of age^13^; however, the experience of imaginary companions is not completely extinct among 9-year-olds. In other words, children may have a stronger tendency than older individuals, such as adults, to treat insubstantial and unreal agents (e.g., imaginary companions or inanimate objects) as real agents. In the present study, the children (but not the adults) might have perceived some sense of “reality” from the AR agent and, thus, avoided passing through the aisle occupied by this “real” agent.

Although Lee *et al*.^4^ reported that information displayed by AR technology has a significant effect on adults’ behavior, the present results did not reveal any significant effects of AR-generated information on pathway selection in adult participants. This discrepancy may be due to differences in the timing of watching the AR information. The previous study measured participants’ responses in real-time during the AR experiences whereas the present study examined participants’ behaviors after they had observed the AR information. Presumably, the real-time presentation of AR information had a stronger effect on behavior in adults than the non-real-time presentation of paradigm AR information in the present study.

On the other hand, a very recent study by Miller, Jun, Herrera, Yu Villa, Welch, and Bailenson^14^ found that behaviors of adult participants are also significantly modulated by the presentation of AR agent even when the behaviors occur just after the presentation of the AR agent. In that experiment, an AR agent was presented to each participant in an experimental room that had two empty chairs. The AR agent walked toward one of the chairs, sat on it, and introduced her/himself (the gender of the AR agent was matched with each participant) to the participant while the participant watched the agent’s behaviors via a head-mounted AR device. Then, the participant was instructed to sit in one of the two chairs: the empty chair or the chair occupied by the AR agent. Most participants chose to sit in the empty chair, even though they had removed the headset before choosing where to sit (i.e., the AR agent was no longer physically invisible). These findings demonstrate that even the non-real-time presentation of AR information can have a significant effect on adult behaviors. However, they appear to be inconsistent with the present results from Experiment 2, which showed no significant effects of the AR character on the behaviors of adult participants.

There are several possible explanations for the inconsistencies between the results of Miller *et al*.^14^ and those from Experiment 2 in the present study. For instance, the difference in the length of the time period between observing the AR character and the selection of the walking behavior might have affected the results. In Miller *et al*.^14^, the participants were asked to choose a chair immediately after taking off the head-mounted display used to present the AR agent whereas the adult participants in the present study typically made their pathway selection and came across the barrier within a few tens of seconds to a minute after the end of the AR character presentation. The longer time period between the observation of the AR character and the choice of behavior in the present study may have weakened the response to the AR character in the adult participants.

Another factor that could have contributed to these discrepancies involves qualitative distinctions between the visual stimuli used in the respective studies. The AR agent used in Miller *et al*.^14^ had a very realistic appearance and spoke to the participants whereas the AR agent in the present study was a human-like but relatively unrealistic cartoon character and did not say anything. Additionally, Miller *et al*.^14^ employed head-mounted equipment that provided an immersive visual experience to present the AR agent whereas the present study used a tablet device held in the hands of the participants to present the AR avatar (it should be noted that Lee *et al*.^4^, who reported a significant effect of AR information on adults’ behavior, also employed a head-mounted device and an avatar with a realistic appearance). Because the use of head-mounted devices for VR/AR in young children is very controversial^15^, head-mounted devices were not used to present AR characters in the present study. It is plausible that the visual stimulus used by Miller *et al*.^14^ (and possibly Lee *et al*.^4^) provided a more real perception and/or feeling of the AR agent than the stimulus used in the present study. Thus, the qualitative differences (e.g., reality of the AR agent) among these studies may have affected the present results of the adult participants. Alternatively, apart from the reality of the AR agent, technical differences in the AR presentation (head-mounted display vs. tablet device) per se might have been related to the discrepancies between the previous studies^4,14^ and the present results. It is likely that an avatar presented by a head-mounted device and one presented by a hand-held device have different effects on human behavior. It will be interesting for future studies to compare the effects of information presented by AR via different technologies (e.g., head-mounted devices, projection mapping, and hand-held devices such as tablets and smart phones) on human behavior.

## Concluding Remarks

Taken together, the present findings indicate that visual information presented via AR technology can have a significant effect on behavior in childhood (approximately 5–10 years of age). Although the adult participants in the present study did not significantly change their behavior in response to the AR character, it is possible that these non-significant results were due to the insufficiently lifelike nature of the AR agent. For example, previous studies have shown that a realistic AR avatar presented by a head-mounted device has a significant effect on the behavior of adult observers (cf.^2,14^). Alternatively, the lack of change in the adults’ behavior in the present study may be specific to AR information presented by hand-held devices, such as a tablet. Thus, future studies should assess the effects of more realistic AR agents (e.g., a more realistic appearance and interactive behaviors) displayed by a variety of AR devices (head-mounted devices and hand-held devices, such as tablets, smartphones, and projection mapping) on observers’ behavior as well as the developmental trajectories of these behavioral effects.
